# Association of the Systemic Immune-Inflammation Index with CCTA-Derived SYNTAX Scores in Patients with Obstructive Coronary Artery Disease

**DOI:** 10.3390/diagnostics16152417

**Published:** 2026-07-31

**Authors:** Davut Unsal Capkan, Mehmet Kaplan

**Affiliations:** 1 Department of Radiology, Medical Point Hospital, Gaziantep 27060, Turkey; 2 Department of Cardiology, Medical Point Hospital, Gaziantep 27060, Turkey; drkaplanmehmet@gmail.com

**Keywords:** systemic immune-inflammation index, platelet-to-lymphocyte ratio, coronary artery disease, SYNTAX score, CCTA, inflammation, coronary anatomical complexity

## Abstract

**Background:** Systemic inflammation plays an important role in the pathogenesis and progression of coronary artery disease (CAD). The systemic immune-inflammation index (SII) reflects inflammatory and immune status, whereas the platelet-to-lymphocyte ratio (PLR) represents a simpler hematological inflammatory index. This study aimed to compare the cross-sectional associations of SII and PLR with coronary computed tomography angiography (CCTA)-derived coronary anatomical complexity among selected patients with established obstructive CAD. **Methods:** In this single-center retrospective cross-sectional study, 197 patients who underwent CCTA were screened. Of these, 104 were excluded, including 68 patients with <50% coronary stenosis, leaving 93 patients with CCTA-defined obstructive CAD (≥50% stenosis) in the final cohort. Patients were categorized as having low, intermediate, or high anatomical complexity according to the CCTA-derived SYNTAX I score. Exploratory ROC analysis assessed discrimination between high (≥33) and lower anatomical complexity within this selected obstructive-CAD cohort. The reduced Firth models underwent internal validation using 1000 bootstrap resamples. **Results:** Eighteen patients had high anatomical complexity. SII correlated more strongly with the SYNTAX I score than PLR (r = 0.59 vs. 0.34). In adjusted linear models, a doubling of SII was associated with a 6.10-point higher SYNTAX I score (95% CI: 3.52–8.68; *p* < 0.001), whereas PLR was not significantly associated (*p* = 0.104). In the Firth models, both SII (per 100-point increase: OR = 1.15, 95% CI: 1.07–1.25) and PLR (per 10-point increase: OR = 1.06, 95% CI: 1.01–1.12) were associated with high complexity. The data-derived SII cut-off of ≥850 yielded 83.3% sensitivity and 76.0% specificity. Optimism-corrected AUCs were 0.82 for age plus SII and 0.70 for age plus PLR. **Conclusions:** In this selected single-center cohort of patients with CCTA-defined obstructive CAD, SII showed a stronger cross-sectional association with concurrently assessed coronary anatomical complexity than PLR. The exploratory ROC estimates apply only to discrimination between high and lower anatomical complexity within the included cohort and do not establish clinical utility.

## 1. Introduction

Coronary artery disease (CAD) remains one of the leading causes of morbidity and mortality worldwide, despite significant advances in diagnostic and therapeutic strategies. Assessment of coronary disease severity and anatomical complexity can inform clinical evaluation and revascularization planning [1,2]. In this context, coronary computed tomography angiography (CCTA) has emerged as a reliable, non-invasive imaging modality that enables detailed visualization of coronary artery anatomy and atherosclerotic burden [3,4,5]. The SYNTAX score, originally developed for invasive coronary angiography, has been successfully adapted for CCTA and is widely used to quantify the anatomical complexity of CAD, incorporating lesion characteristics such as location, length, calcification, bifurcation, and total occlusion [6,7,8].

Although the SYNTAX score provides valuable anatomical information, it does not reflect the biological activity of atherosclerosis, particularly the inflammatory processes underlying plaque formation and progression. A growing body of evidence indicates that systemic inflammation plays a central role in all stages of atherosclerosis, from endothelial dysfunction to plaque instability and rupture [9,10,11,12]. Therefore, anatomical assessment tools and systemic inflammatory biomarkers may provide complementary information about coronary disease burden and systemic inflammatory status.

Among inflammation-related biomarkers, measures derived from routine complete blood counts have gained attention because of their accessibility and low cost. These include the neutrophil-to-lymphocyte ratio (NLR), platelet-to-lymphocyte ratio (PLR), and individual platelet, neutrophil, and lymphocyte counts. NLR reflects the relative predominance of innate inflammatory activity over lymphocyte-mediated immune regulation, whereas PLR combines platelet-related thromboinflammatory activity with relative lymphopenia. C-reactive protein (CRP), although not derived from the complete blood count, is another readily available marker reflecting the hepatic acute-phase response. These measures capture related but partly distinct components of systemic inflammation and have been associated with cardiovascular disease severity and outcomes [13,14,15,16].

The systemic immune-inflammation index (SII), calculated as platelet count × neutrophil count/lymphocyte count, combines three circulating cellular components and has been investigated as a composite measure of inflammatory, thrombotic, and immune activity [17,18]. Several studies have reported associations between elevated SII and the severity or prognosis of CAD [19,20,21]. However, whether this composite index provides information beyond that conveyed by simpler hematological indices remains uncertain.

PLR was selected as the primary comparator because it is an established complete blood count-derived index in cardiovascular research and shares the platelet and lymphocyte components included in SII [13,15]. Mathematically, SII can be expressed as PLR multiplied by the neutrophil count. Thus, comparing SII with PLR provides a focused assessment of whether inclusion of the neutrophil component is associated with a stronger relationship with coronary anatomical complexity. Nevertheless, this shared mathematical structure also limits interpretation: a stronger association of SII may primarily reflect the contribution of neutrophilia or mathematical weighting rather than indicate a fundamentally superior composite inflammatory construct. NLR, CRP, and individual blood-cell counts remain relevant alternative comparators; however, the present study was designed as a focused head-to-head comparison of SII and PLR rather than a comprehensive evaluation of all available inflammatory biomarkers.

Although observational studies have linked higher SII values to coronary disease severity and cardiovascular outcomes [22,23,24,25], direct comparisons of SII and PLR in relation to CCTA-derived coronary anatomical complexity remain limited. We therefore compared their cross-sectional associations with CCTA-derived SYNTAX scores in patients with established obstructive CAD.

## 2. Materials and Methods

### 2.1. Study Design and Population

This single-center, retrospective, observational, cross-sectional study was conducted at Gaziantep Medical Point Hospital to assess coronary anatomical complexity among patients with CCTA-defined obstructive CAD. The study was conducted in accordance with the ethical principles of the Declaration of Helsinki. The study protocol was reviewed and approved by the Gaziantep City Hospital Non-Interventional Clinical Research Ethics Committee (Approval No: 354/2025; Date: 17 December 2025).

The institutional PACS database was searched to identify all patients who underwent clinically indicated CCTA between 1 August 2022 and 30 October 2025. No sampling or case-matching procedure was applied. The clinical indications for CCTA included evaluation of chest pain or anginal-equivalent symptoms suggestive of CAD, further assessment following abnormal or inconclusive non-invasive ischemia testing, and anatomical evaluation of suspected CAD in patients with an intermediate pre-test probability. Clinical, laboratory, and imaging data were obtained from the hospital electronic medical records and PACS database.

Patients were eligible for the present analysis if they were aged ≥18 years, had at least one coronary lesion producing ≥50% diameter stenosis on CCTA, had images of sufficient quality for SYNTAX score calculation, and had complete clinical, demographic, and laboratory data, including platelet, neutrophil, and lymphocyte counts obtained within ±15 days of CCTA. Patients with <50% coronary stenosis were excluded because the prespecified objective was to assess variation in anatomical complexity within patients with obstructive CAD. Additional exclusion criteria were inadequate image quality or substantial motion artifacts, incomplete hematological parameters, unavailable clinical records, severe structural heart disease, active infection, malignancy, systemic inflammatory disease, and pregnancy. Consequently, the resulting cohort does not represent an unselected population undergoing CCTA and cannot be used to assess whether SII distinguishes patients with and without obstructive CAD.

### 2.2. Data Collection and Measurements

Clinical, demographic, laboratory, and imaging data were retrospectively obtained from the hospital’s electronic medical records and picture archiving and communication system (PACS). Demographic variables included age and sex, while clinical data comprised comorbid conditions such as hypertension, diabetes mellitus, dyslipidemia, smoking status, and current medication use (including statins, angiotensin-converting enzyme inhibitors, beta-blockers, and antiplatelet agents). Laboratory parameters were derived from routine complete blood count analyses and included platelet, neutrophil, and lymphocyte counts, as well as hemoglobin, hematocrit, and mean corpuscular volume values. To ensure temporal consistency, only laboratory measurements obtained within ±15 days of the CCTA examination were included.

Inflammatory indices were calculated directly from the absolute blood-cell counts reported by the hematology analyzer. Platelet, neutrophil, and lymphocyte counts were expressed in ×10^9^/L, and their numerical values were entered into the corresponding formulas. SII was calculated as platelet count × neutrophil count/lymphocyte count, whereas PLR was calculated as platelet count/lymphocyte count. Both SII and PLR were reported as unitless indices. No additional multiplication, division, or rescaling was applied. Accordingly, the exploratory SII cut-off was reported as 850 without a unit.

Medication exposure was defined according to active treatment documented at the time of blood sampling and CCTA. Recorded medications included statins, angiotensin-converting enzyme inhibitors, beta-blockers, and antiplatelet agents. Their distributions across the low, intermediate, and high SYNTAX I score categories were compared using the chi-square test or Fisher’s exact test, as appropriate.

CCTA examinations were performed using a 640-slice multidetector computed tomography scanner (Aquilion ONE, Canon Medical Systems Corporation [formerly Toshiba Medical Systems], Otawara, Japan) with standardized ECG-gated acquisition protocols. Image acquisition was performed using prospective or retrospective ECG-gating according to heart rate and clinical indications. Prior to imaging, patients with elevated heart rates received rate-controlling medication when appropriate to achieve optimal image quality. Intravenous non-ionic iodinated contrast material (60–80 mL) was administered through an antecubital vein at a flow rate of approximately 5 mL/s, followed by a saline flush. Imaging parameters included detector collimation of 0.5 mm, tube voltage of 100–120 kVp, tube current modulation according to body habitus, gantry rotation time of 0.275–0.35 s, and reconstructed slice thickness of 0.6 mm. Image acquisition was synchronized with the cardiac cycle to minimize motion artifacts and ensure high spatial resolution. All datasets were transferred to a dedicated workstation for post-processing and analysis. Multiplanar reformations, maximum intensity projections, and three-dimensional volume-rendered images were generated for comprehensive evaluation of coronary artery anatomy. Only scans with sufficient diagnostic quality were included in the analysis, whereas examinations with significant motion artifacts or inadequate visualization were excluded.

The CCTA-derived anatomical SYNTAX I score was calculated using the official web-based SYNTAX Score Calculator, version 2.28 (www.syntaxscore.org; accessed on 17 January 2026). All coronary lesions producing ≥50% diameter stenosis in vessels ≥ 1.5 mm in diameter were evaluated. The scored lesion characteristics included coronary dominance, anatomical location, total occlusion, bifurcation or trifurcation involvement, aorto-ostial location, severe tortuosity, lesion length > 20 mm, heavy calcification, thrombus, and diffuse disease or small-vessel involvement. Individual lesion scores were summed to obtain the total anatomical SYNTAX I score. Patients were categorized as having low (0–22), intermediate (23–32), or high (≥33) anatomical complexity.

The original 2013 SYNTAX Score II 4-year mortality model was calculated using the official online SYNTAX Score II calculator. The variables entered were the CCTA-derived anatomical SYNTAX I score, age, sex, creatinine clearance, left ventricular ejection fraction, presence of unprotected left main coronary artery disease, chronic obstructive pulmonary disease, and peripheral vascular disease. Creatinine clearance was calculated using the Cockcroft–Gault equation. All required clinical variables were available for the included patients; no imputation, default values, or substitution procedures were used. The calculator generated separate predicted 4-year all-cause mortality estimates for PCI and CABG. Clinical variables were entered only after the CCTA-derived anatomical score had been finalized.

Previous studies have demonstrated the feasibility and reproducibility of calculating anatomical SYNTAX scores from CCTA and have reported agreement between CCTA- and invasive angiography-based assessments [1,6,7]. Nevertheless, the original SYNTAX Score II was developed primarily using invasive coronary angiography in patients with complex multivessel or left main disease. Therefore, the CCTA-derived SYNTAX II estimates in the present study were used as exploratory research measures and were not used to make clinical revascularization decisions.

CCTA-derived anatomical SYNTAX scores were assessed without access to SII, PLR, other laboratory measurements, or calculated SYNTAX II results. For reproducibility analysis, 30 randomly selected CCTA examinations were independently evaluated by a second radiologist with more than 10 years of cardiovascular imaging experience, who was blinded to the primary reader’s measurements and to the patients’ laboratory and clinical data. The primary reader repeated the anatomical assessment after a four-week interval without access to the original scores. Interobserver reliability was evaluated using a two-way random-effects, absolute-agreement, single-measure ICC. Intraobserver reliability was evaluated using a two-way mixed-effects, absolute-agreement, single-measure ICC. ICC values were interpreted as poor (<0.50), moderate (0.50–0.75), good (0.75–0.90), or excellent (>0.90), and 95% confidence intervals were reported.

### 2.3. Outcome Measures

The primary outcome was the strength of the cross-sectional associations of SII and PLR with CCTA-derived SYNTAX scores among patients with established obstructive CAD. Secondary outcomes included comparisons of SII and PLR across low-, intermediate-, and high-complexity SYNTAX categories and exploratory evaluation of their ability to distinguish patients with high anatomical complexity (SYNTAX score ≥ 33) from those with low or intermediate complexity within the selected cohort. These analyses were not intended to assess the ability of either index to identify the presence of obstructive CAD in an unselected CCTA population.

Because this was a retrospective study, no a priori sample-size calculation was performed. All eligible consecutive patients identified during the predefined study period were included. The final cohort comprised 93 patients, of whom 18 had a high SYNTAX score (19.4%; exact 95% CI: 11.9–28.9%). Given the limited number of outcome events, model complexity was restricted to reduce the risk of overfitting. The original six-parameter model would have provided only 3.0 events per parameter. Therefore, the revised adjusted analyses were limited to two prespecified parameters per model, corresponding to nine events per parameter. The resulting analyses should nevertheless be considered exploratory because of the limited sample size and number of events.

### 2.4. Statistical Analysis

All statistical analyses were performed using IBM SPSS Statistics version 27.0 (IBM Corp., Armonk, NY, USA) and R software (version 4.3.0; R Foundation for Statistical Computing, Vienna, Austria). Continuous variables were expressed as mean ± standard deviation (SD) or median (interquartile range, IQR) according to data distribution, while categorical variables were presented as frequencies and percentages. Comparisons across the low, intermediate, and high SYNTAX I score categories were performed using one-way analysis of variance or the Kruskal–Wallis test, as appropriate. Significant overall comparisons were followed by Tukey’s test after analysis of variance or Dunn’s test with Holm adjustment after the Kruskal–Wallis test. Categorical variables were compared using the chi-square test or Fisher’s exact test, as appropriate. Associations of SII and PLR with the CCTA-derived SYNTAX I and exploratory SYNTAX II PCI and CABG estimates were assessed using Pearson or Spearman correlation coefficients according to variable distribution. Univariable linear regression was used to examine associations with the continuous CCTA-derived SYNTAX I score. Because SII and PLR were right-skewed, they were log_2_-transformed; their coefficients therefore represent the mean difference in SYNTAX I score associated with a doubling of the corresponding index. To maintain model parsimony, two reduced multivariable linear regression models were constructed. Model 1 included age, ejection fraction, creatinine clearance, and log_2_-transformed SII, whereas Model 2 included the same clinical covariates and log_2_-transformed PLR. These clinical covariates were selected on clinical grounds, and no automated stepwise selection was used. SII and PLR were not entered into the same model because SII is mathematically equivalent to PLR multiplied by the neutrophil count. Unstandardized coefficients, standard errors, 95% confidence intervals, standardized coefficients, and *p*-values were reported. Model fit was summarized using R^2^, adjusted R^2^, and root mean squared error. VIF values were reported for every predictor. Linearity, residual normality, homoscedasticity, and influential observations were assessed using partial-residual plots, Q–Q plots and the Shapiro–Wilk test, residual-versus-fitted plots and the Breusch–Pagan test, and Cook’s distance, respectively. Because only 18 patients had a high SYNTAX I score (≥33), reduced Firth penalized logistic regression models were used. Model 1 included age and SII per 100-point increase, whereas Model 2 included age and PLR per 10-point increase. Each model therefore contained two prespecified parameters, corresponding to nine outcome events per parameter. Penalized odds ratios with profile-likelihood 95% confidence intervals were reported. SII and PLR were evaluated in separate models because of their mathematical overlap. Receiver operating characteristic (ROC) analysis was performed as an exploratory assessment of the ability of SII and PLR to distinguish high from lower anatomical complexity within the selected obstructive-CAD cohort. AUC values were reported with 95% confidence intervals and compared using DeLong’s test. Cut-offs were selected using the Youden index and were considered data-derived rather than prespecified clinical thresholds. Sensitivity and specificity were reported with 95% exact binomial confidence intervals together with the corresponding two-by-two classification counts. Marker-only ROC and cut-off estimates were considered exploratory and were not interpreted as evidence of incremental clinical utility. Internal validation of the reduced Firth logistic regression models was performed using 1000 bootstrap resamples. In each resample, the model was refitted, and its performance was evaluated in both the bootstrap sample and the original dataset. Mean optimism was subtracted from the apparent performance to obtain optimism-corrected AUC, calibration slope, and Brier score estimates. NRI and IDI analyses were not retained because no prespecified risk categories or adequately powered reference clinical model were available. To assess potential temporal variability, a sensitivity analysis was performed after restricting the cohort to patients whose complete blood count was obtained on the same day as or within five days of CCTA. Correlations of SII and PLR with the CCTA-derived SYNTAX scores were repeated in this restricted subgroup. All analyses were considered exploratory and hypothesis-generating, and two-sided *p*-values < 0.05 were considered statistically significant.

## 3. Results

**Figure 1:**
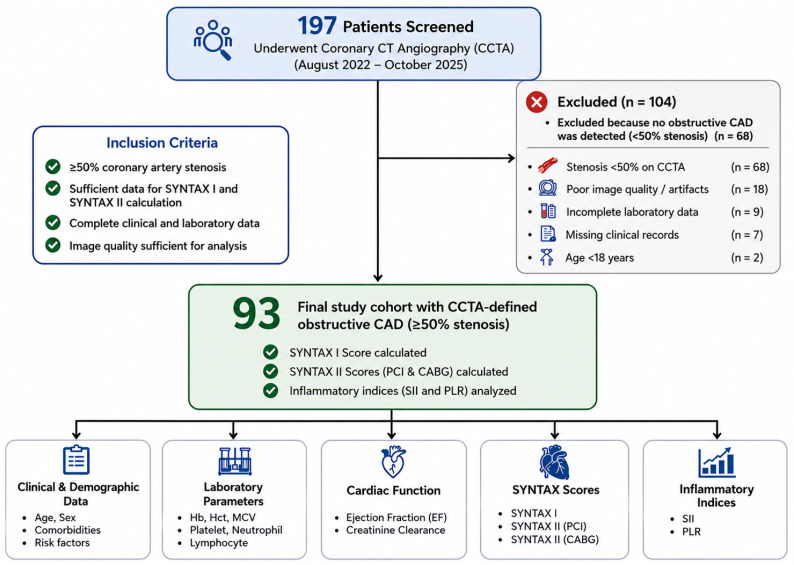
Flowchart of patient selection. CCTA, coronary computed tomography angiography; CAD, coronary artery disease; SII, systemic immune-inflammation index; PLR, platelet-to-lymphocyte ratio; PCI, percutaneous coronary intervention; CABG, coronary artery bypass grafting.

A total of 197 patients who underwent clinically indicated CCTA between August 2022 and October 2025 were screened. Of these, 68 patients with <50% coronary stenosis were excluded. An additional 36 patients were excluded because of inadequate image quality or motion artifacts (*n* = 18), incomplete laboratory data (*n* = 9), unavailable clinical records (*n* = 7), or age <18 years (*n* = 2). The final analysis therefore included 93 selected patients with CCTA-defined obstructive CAD (≥50% stenosis) and complete clinical, laboratory, and imaging data. Accordingly, the study population represents a subgroup with established obstructive disease rather than a general population referred for CCTA (Figure 1).

The demographic, clinical, and laboratory characteristics of patients included in the final analysis and those excluded because of <50% coronary stenosis are compared in Supplementary Table S1. Compared with the excluded patients, those included in the obstructive-CAD cohort were older and had higher neutrophil counts, SII, and PLR values, as well as lower ejection fraction and creatinine clearance. No statistically significant differences were observed in sex, hypertension, diabetes mellitus, smoking, dyslipidemia, COPD, peripheral vascular disease, hemoglobin, hematocrit, platelet count, or lymphocyte count (Supplementary Table S1).

The baseline demographic, clinical, and laboratory characteristics of the study population (*n* = 93) are summarized in Table 1. The mean age of the patients was 62.4 ± 10.8 years, and 64 patients (68.8%) were male. Hypertension was present in 58 patients (62.4%), diabetes mellitus in 36 (38.7%), smoking in 41 (44.1%), dyslipidemia in 49 (52.7%), chronic obstructive pulmonary disease in 14 (15.1%), and peripheral vascular disease in 11 (11.8%). The mean hemoglobin level was 13.6 ± 1.5 g/dL, hematocrit was 40.8 ± 4.3%, and mean corpuscular volume was 87.9 ± 6.8 fL. Median platelet count was 256 (210–305) × 10^9^/L, neutrophil count was 4.9 (3.8–6.2) × 10^9^/L, and lymphocyte count was 1.7 (1.3–2.2) × 10^9^/L. The mean EF was 53.6 ± 8.9%, and mean creatinine clearance was 79.4 ± 22.1 mL/min. The median SII value was 650 (480–890), and the median PLR was 150 (115–195). The mean SYNTAX I score was 21.8 ± 9.6. The median SYNTAX II predicted mortality was 2.9% (1.8–4.6) for PCI and 2.3% (1.5–3.7) for CABG (Table 1).

**Figure 2:**
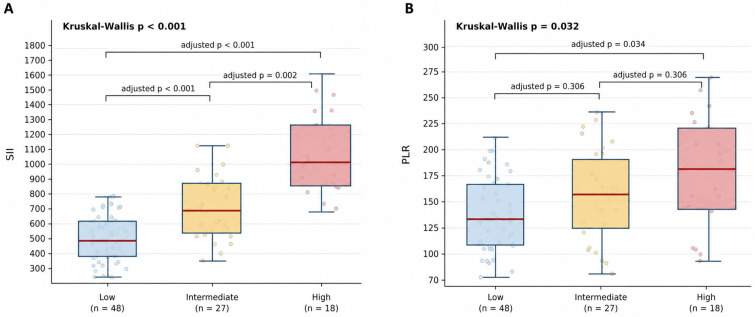
Distribution of SII and PLR across CCTA-derived SYNTAX I score categories. (**A**) SII values in the low-, intermediate-, and high-complexity groups. (**B**) PLR values in the corresponding groups. The central lines indicate medians, boxes represent interquartile ranges, whiskers extend to 1.5 times the interquartile range, and individual points represent patients. Overall differences were evaluated using the Kruskal–Wallis test. Pairwise comparisons were performed using Dunn’s post hoc test with Holm adjustment for multiple testing; the displayed *p*-values are adjusted *p*-values. SII, systemic immune-inflammation index; PLR, platelet-to-lymphocyte ratio; CCTA, coronary computed tomography angiography.

The comparison of clinical and laboratory parameters according to the SYNTAX I score categories is presented in Table 2. Overall differences were observed for age, diabetes mellitus, platelet, neutrophil and lymphocyte counts, SII, PLR, ejection fraction, and creatinine clearance. In the adjusted pairwise analyses, patients in the high-complexity group were older and had higher platelet counts, lower ejection fraction, and lower creatinine clearance than those in the low-complexity group. Neutrophil counts increased, and lymphocyte counts decreased significantly across the three complexity groups. SII differed significantly between all three groups, whereas PLR differed significantly only between the low- and high-complexity groups. Although the overall comparison for diabetes mellitus was significant, none of the pairwise comparisons remained significant after adjustment for multiple testing (Table 2, Figure 2). The distribution of cardiovascular medication use across the SYNTAX I score categories is presented in Supplementary Table S2.

Correlation analysis demonstrated a strong positive association between SII and SYNTAX I score (r = 0.59, *p* < 0.001), as well as with SYNTAX II scores for both PCI (r = 0.58, *p* < 0.001) and CABG (r = 0.55, *p* < 0.001). In contrast, PLR showed only a modest correlation with SYNTAX I (r = 0.34, *p* = 0.002), SYNTAX II PCI (r = 0.29, *p* = 0.008), and SYNTAX II CABG (r = 0.27, *p* = 0.014).

The median absolute interval between blood sampling and CCTA was 2 days (IQR: 0–5; range: 0–15 days). Blood sampling was performed on the same day as CCTA in 41 patients (44.1%) and within five days in 72 patients (77.4%). In the sensitivity analysis restricted to measurements obtained within five days, SII remained correlated with the SYNTAX I, SYNTAX II PCI, and SYNTAX II CABG scores (r = 0.60, r = 0.58, and r = 0.55, respectively; all *p* < 0.001). The corresponding correlations for PLR were r = 0.33 (*p* = 0.005), r = 0.30 (*p* = 0.010), and r = 0.28 (*p* = 0.017), respectively (Supplementary Table S3).

In the reduced multivariable linear regression models, age, ejection fraction, creatinine clearance, and SII were significantly associated with the CCTA-derived SYNTAX I score in Model 1. A doubling of SII was associated with a 6.10-point increase in the SYNTAX I score (B = 6.10, 95% CI: 3.52 to 8.68; standardized β = 0.46; *p* < 0.001). Age was positively associated with the SYNTAX I score (B = 0.14, 95% CI: 0.03 to 0.25; *p* = 0.019), whereas ejection fraction (B = −0.24, 95% CI: −0.43 to −0.05; *p* = 0.014) and creatinine clearance (B = −0.073, 95% CI: −0.139 to −0.007; *p* = 0.029) were negatively associated with the score. Model 1 had an R^2^ of 0.48 and an adjusted R^2^ of 0.46. In Model 2, age, ejection fraction, and creatinine clearance remained significantly associated with the SYNTAX I score, whereas PLR was not significantly associated after adjustment (B = 2.35 per doubling, 95% CI: −0.49 to 5.19; standardized β = 0.15; *p* = 0.104). Model 2 had an R^2^ of 0.35 and an adjusted R^2^ of 0.32. VIF values ranged from 1.08 to 1.24 (Table 3).

The reduced Firth penalized logistic regression results are presented in Table 4. In Model 1, the penalized OR was 1.04 per one-year increase in age (95% CI: 1.00–1.09, *p* = 0.048) and 1.15 per 100-point increase in SII (95% CI: 1.07–1.25, *p* < 0.001). In Model 2, the penalized OR was 1.05 per one-year increase in age (95% CI: 1.01–1.10, *p* = 0.021) and 1.06 per 10-unit increase in PLR (95% CI: 1.01–1.12, *p* = 0.026) (Table 4).

Bootstrap internal validation results are presented in Supplementary Table S4. For Model 1, which included age and SII, the apparent AUC was 0.86, with an estimated optimism of 0.04 and an optimism-corrected AUC of 0.82 (bootstrap 95% CI: 0.72–0.90). The optimism-corrected calibration slope was 0.83, and the corrected Brier score was 0.116. For Model 2, which included age and PLR, the apparent and optimism-corrected AUC values were 0.75 and 0.70, respectively (bootstrap 95% CI: 0.57–0.82). The optimism-corrected calibration slope was 0.75, and the corrected Brier score was 0.144 (Supplementary Table S4).

**Figure 3:**
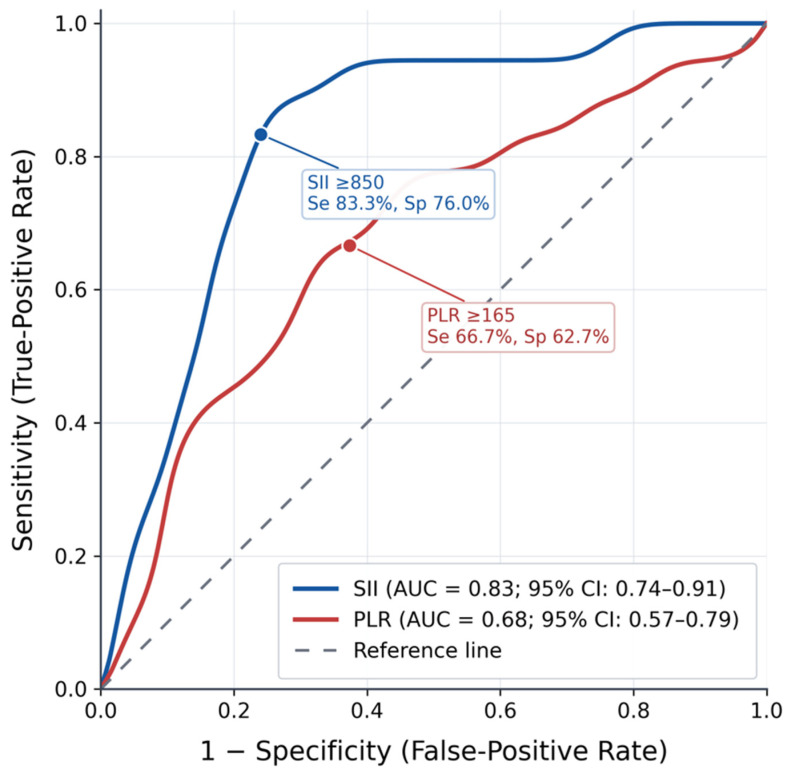
ROC curves comparing the discriminative performance of SII and PLR for distinguishing high from lower coronary anatomical complexity within the obstructive-CAD cohort. ROC, receiver operating characteristic; AUC, area under the curve; SII, systemic immune-inflammation index; PLR, platelet-to-lymphocyte ratio; CAD, coronary artery disease.

In the exploratory ROC analysis, SII yielded an AUC of 0.83 (95% CI: 0.74–0.91; *p* < 0.001). At the data-derived cut-off of ≥850, sensitivity was 83.3% (15/18; exact 95% CI: 58.6–96.4), and specificity was 76.0% (57/75; exact 95% CI: 64.7–85.1). PLR yielded an AUC of 0.68 (95% CI: 0.57–0.79; *p* = 0.006). At the data-derived cut-off of ≥165, sensitivity was 66.7% (12/18; exact 95% CI: 41.0–86.7), and specificity was 62.7% (47/75; exact 95% CI: 50.7–73.6). The difference between the AUC values was 0.15 (95% CI: 0.03–0.27; DeLong *p* = 0.012) (Figure 3). The corresponding two-by-two classification results are provided in Supplementary Table S5.

**Figure 4:**
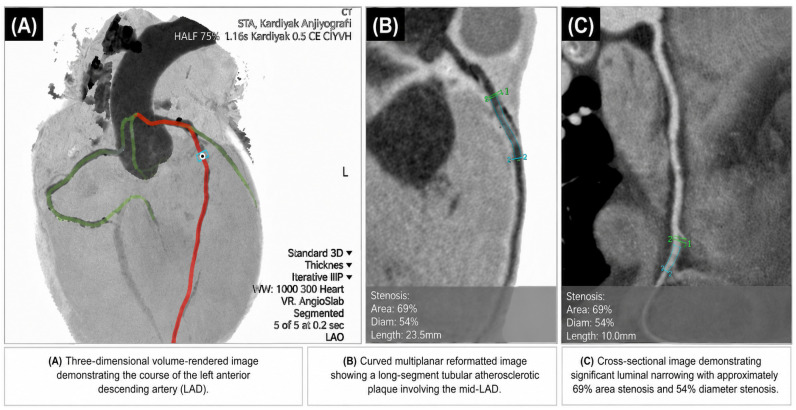
Representative CCTA findings of a high-SYNTAX coronary lesion. (**A**) Three-dimensional volume-rendered image demonstrating the coronary artery anatomy and the course of the left anterior descending artery (LAD). (**B**) Curved multiplanar reformatted image showing a long-segment tubular atherosclerotic plaque involving the mid-LAD, with approximately 69% area stenosis, 54% diameter stenosis, and a lesion length of 23.5 mm. (**C**) Curved multiplanar reformatted image demonstrating an additional stenotic segment within the LAD, with a lesion length of approximately 10.0 mm. The presence of multiple long-segment stenotic lesions contributes to increased coronary artery disease complexity as reflected by the SYNTAX score.

Interobserver agreement for SYNTAX score assessment was excellent (ICC = 0.92, 95% CI: 0.87–0.96), while intraobserver agreement was also excellent (ICC = 0.95, 95% CI: 0.91–0.98). Representative CCTA images illustrating coronary lesions with high anatomical complexity are shown in Figure 4. Three-dimensional volume-rendered imaging demonstrated the coronary artery anatomy and the course of the LAD (Figure 4A). Curved multiplanar reformatted imaging identified a long-segment tubular atherosclerotic plaque involving the mid-LAD with 69% area stenosis, 54% diameter stenosis, and a lesion length of 23.5 mm (Figure 4B). An additional stenotic LAD segment with a lesion length of 10.0 mm was also demonstrated on curved multiplanar reformatted imaging (Figure 4C).

## 4. Discussion

In this selected cohort of patients with CCTA-defined obstructive CAD, SII showed a stronger cross-sectional association with coronary anatomical complexity than PLR. SII had stronger correlations with the SYNTAX I and exploratory SYNTAX II estimates and remained associated with the continuous SYNTAX I score after adjustment. Although both indices were associated with high anatomical complexity in the reduced Firth models, exploratory ROC analysis showed greater discrimination for SII. These findings describe concurrent associations within the included cohort and do not establish diagnostic, prognostic, or incremental clinical utility.

Our findings are consistent with previous studies reporting the association between systemic inflammatory markers and coronary artery disease severity. Yang et al. demonstrated that SII was significantly associated with adverse clinical outcomes in patients with coronary artery disease, highlighting its prognostic relevance [19]. Liu et al. reported that SII was positively correlated with the severity of coronary stenosis, suggesting that it reflects the extent of atherosclerotic burden [21]. More recently, Wang et al. showed that SII was a strong predictor of coronary lesion severity in patients with diabetes mellitus, further supporting its role as a marker of vascular inflammation and complexity [20]. In addition, Candemir et al. demonstrated a significant association between SII and SYNTAX score, indicating that higher SII levels are linked with more complex coronary artery disease [26]. Altunova et al. reported that SII was an independent predictor of residual SYNTAX score, supporting its role in reflecting coronary anatomical complexity [27]. Zhao et al. showed that SII has superior predictive performance compared to PLR in assessing coronary severity, which is in line with our findings. Meta-analytic evidence also indicates that elevated SII levels are associated with increased cardiovascular risk and disease burden [28]. In line with these findings, our study extends the existing literature by demonstrating that SII is not only associated with disease presence or severity but also closely reflects anatomical complexity as quantified by SYNTAX scoring derived from CCTA.

Compared with SII, PLR showed weaker correlations with the SYNTAX scores and was not significantly associated with the SYNTAX I score after multivariable adjustment. This difference may be related to the inclusion of the neutrophil count in the SII formula. Because SII is mathematically equivalent to PLR multiplied by the neutrophil count, its stronger association with coronary complexity may primarily reflect the contribution of neutrophilia or mathematical weighting rather than the superiority of a fundamentally distinct composite inflammatory construct. The progressive increase in neutrophil counts across the SYNTAX categories in the present cohort is consistent with this interpretation. Pruc et al. emphasized that although PLR is associated with adverse outcomes in acute coronary syndromes, its predictive performance is modest and may be inferior to composite indices [13]. Tudurachi et al. similarly highlighted that single-ratio markers such as PLR may be less informative than more integrative indices combining multiple immune pathways [15]. NLR, CRP, and individual blood-cell counts may provide complementary or component-specific inflammatory information; however, they were not evaluated as primary head-to-head comparators in the present analysis. Therefore, our findings demonstrate only that SII had a stronger association with coronary anatomical complexity than PLR within this dataset; they do not establish the general superiority of SII over other readily available inflammatory markers. Future adequately powered studies should directly compare SII with NLR, CRP, and its individual cellular components and validate the resulting models in independent populations.

The stronger association observed for SII should be interpreted in light of its mathematical relationship with PLR. SII is equivalent to PLR multiplied by the neutrophil count; therefore, its stronger association may partly reflect the contribution of neutrophilia or mathematical weighting rather than the superiority of a fundamentally distinct inflammatory construct. Biologically, neutrophils contribute to vascular inflammation and plaque instability, platelets participate in thromboinflammatory activity, and lymphocytes have regulatory immune functions [9,10]. The progressive increase in neutrophil counts across the SYNTAX categories is consistent with this interpretation but does not establish a causal mechanism. Because NLR, CRP, and the individual cellular components were not evaluated as primary head-to-head comparators, no conclusion can be drawn regarding the general superiority of SII over other inflammatory markers.

An additional consideration is that contemporary CCTA techniques can provide not only anatomical information but also imaging-derived markers of vascular inflammation. The perivascular fat attenuation index (pFAI) captures CT attenuation changes in perivascular adipose tissue associated with local vascular inflammatory signalling. Savo et al. reported that pFAI may provide prognostic information beyond conventional anatomical and calcium-based assessments in patients with coronary artery disease [29]. More recently, Sansonetti et al. reported that CTA-derived pericoronary FAI predicted allograft rejection and cardiovascular events in heart-transplant recipients, extending its potential relevance to another cardiovascular population [30]. In contrast, SII reflects circulating cellular components that may change in response to transient systemic conditions. SII and perivascular fat attenuation should therefore be considered potentially complementary rather than interchangeable measures. Prospective studies combining these markers may clarify whether they provide non-redundant information about coronary anatomical complexity.

An important finding of this study is the stepwise increase in SII across SYNTAX score categories, indicating a graded relationship between systemic inflammation and anatomical disease burden. This trend supports the concept that inflammatory activity parallels the progression of coronary atherosclerosis. Dziedzic et al. reported similar findings, demonstrating that higher SII values were associated with more advanced coronary lesions and increased risk of acute coronary syndromes [22]. In addition, Li et al. showed that SII could predict clinical prognosis in patients with newly diagnosed coronary artery disease, reinforcing its potential role in risk stratification [23]. Our results further contribute to this evidence by linking SII to a widely accepted anatomical scoring system, thereby enhancing its clinical relevance.

The ROC findings should be interpreted as exploratory discrimination between high and lower anatomical complexity within a selected obstructive-CAD cohort. They do not represent diagnostic performance for detecting obstructive CAD because patients without obstructive disease were excluded. Moreover, the cut-offs were derived and evaluated in the same small dataset and may therefore be optimistic. The study also did not assess incremental value over an established clinical model or evaluate clinical usefulness using decision-curve analysis. External validation in larger, prospectively defined cohorts is required before these estimates or cut-offs can be considered for clinical application.

Unlike previous studies that focused primarily on the presence or severity of CAD and subsequent clinical outcomes [19,20,21], the present study directly compared the cross-sectional associations of SII and PLR with CCTA-derived coronary anatomical complexity. Across correlation, reduced multivariable regression, and exploratory ROC analyses, SII showed a stronger association with anatomical complexity and greater discrimination between high and lower complexity than PLR. However, these findings should not be equated with clinical usefulness because the estimates and cut-offs were derived from a small, selected cohort and were not evaluated against an established clinical model. Therefore, the results should be considered hypothesis-generating and do not support the use of SII for screening, pre-test risk stratification, or treatment decisions. Prospective studies incorporating calibration, decision-curve analysis, and independent external validation are required before any clinical application can be considered.

Several limitations should be acknowledged. First, the single-center, retrospective, cross-sectional design limits causal inference and generalizability. The inclusion of only patients with CCTA-defined obstructive CAD resulted in spectrum restriction and possible selection bias. Consequently, the study cannot determine whether SII distinguishes obstructive CAD from non-obstructive disease or the absence of CAD. The cross-sectional design also precludes assessment of temporal direction, and reverse association cannot be excluded. Although patients with active infection, malignancy, and systemic inflammatory disease were excluded, residual confounding from unrecognized inflammatory activity, comorbidities, and medication use may remain. Second, the sample size was limited, particularly in the high-complexity group. Firth penalization and bootstrap internal validation reduced small-sample bias and quantified optimism but cannot replace validation in an independent cohort. The ROC cut-offs were derived and evaluated in the same dataset. SII and PLR were calculated from a single blood measurement, although a narrower-interval sensitivity analysis was performed. In addition, the original SYNTAX II model was developed primarily using invasive angiography in patients with complex multivessel or left main disease; therefore, the CCTA-derived SYNTAX II estimates should be regarded as exploratory. Finally, CCTA provided anatomical rather than functional assessment of coronary lesions.

## 5. Conclusions

In conclusion, SII showed a stronger association with CCTA-derived coronary anatomical complexity than PLR within this selected cohort of patients with established obstructive CAD. SII also demonstrated greater exploratory discrimination for high anatomical complexity; however, these cross-sectional findings do not establish causality, diagnostic or prognostic performance, incremental clinical value, or clinical usefulness. SII should therefore be considered a hypothesis-generating correlate of coronary anatomical complexity rather than a validated biomarker for screening, risk stratification, or treatment decisions. Confirmation in adequately powered prospective multicenter cohorts using prespecified models and independent external validation is required.

## Figures and Tables

**Table 1:** Baseline demographic, clinical, and laboratory characteristics of the study population (*n* = 93).

Variable	Value Mean ± SD/Median (IQR)/*n* (%)
Age (years)	62.4 ± 10.8
Male sex, *n* (%)	64 (68.8%)
Hypertension, *n* (%)	58 (62.4%)
Diabetes mellitus, *n* (%)	36 (38.7%)
Smoking, *n* (%)	41 (44.1%)
Dyslipidemia, *n* (%)	49 (52.7%)
COPD, *n* (%)	14 (15.1%)
Peripheral vascular disease, *n* (%)	11 (11.8%)
Hemoglobin (g/dL)	13.6 ± 1.5
Hematocrit (%)	40.8 ± 4.3
MCV (fL)	87.9 ± 6.8
Platelet (×10^9^/L)	256 (210–305)
Neutrophil (×10^9^/L)	4.9 (3.8–6.2)
Lymphocyte (×10^9^/L)	1.7 (1.3–2.2)
Ejection fraction (%)	53.6 ± 8.9
Creatinine clearance (mL/min)	79.4 ± 22.1
SII	650 (480–890)
PLR	150 (115–195)
SYNTAX I score	21.8 ± 9.6
SYNTAX II (PCI mortality, %)	2.9 (1.8–4.6)
SYNTAX II (CABG mortality, %)	2.3 (1.5–3.7)
PCI recommended, *n* (%)	58 (62.4%)
CABG recommended, *n* (%)	35 (37.6%)

SD, standard deviation; IQR, interquartile range; COPD, chronic obstructive pulmonary disease; MCV, mean corpuscular volume; SII, systemic immune-inflammation index; PLR, platelet-to-lymphocyte ratio; SYNTAX, Synergy Between PCI With Taxus and Cardiac Surgery; PCI, percutaneous coronary intervention; CABG, coronary artery bypass grafting.

**Table 2:** Comparison of inflammatory indices and clinical parameters according to SYNTAX I score categories (Groups: Low (0–22), Intermediate (23–32), High (≥33)).

Variable	Low (*n* = 48)	Intermediate (*n* = 27)	High (*n* = 18)	Overall *p*-Value	Significant Pairwise Comparisons, Adjusted *p*
Age (years)	59.8 ± 9.6	63.7 ± 10.4	67.2 ± 11.1	0.021	Low vs. High, *p* = 0.019
Male sex, *n* (%)	30 (62.5%)	19 (70.4%)	15 (83.3%)	0.18	—
Hypertension, *n* (%)	26 (54.2%)	18 (66.7%)	14 (77.8%)	0.09	—
Diabetes mellitus, *n* (%)	14 (29.2%)	12 (44.4%)	10 (55.6%)	0.048	
Smoking, *n* (%)	19 (39.6%)	13 (48.1%)	9 (50.0%)	0.52	—
Dyslipidemia, *n* (%)	22 (45.8%)	15 (55.6%)	12 (66.7%)	0.17	—
Platelet (×10^9^/L)	240 (200–280)	265 (220–310)	285 (240–330)	0.041	Low vs. High, *p* = 0.039
Neutrophil (×10^9^/L)	4.3 (3.5–5.2)	5.2 (4.1–6.5)	6.1 (4.9–7.3)	<0.001	Low vs. Intermediate, *p* = 0.018; Low vs. High, *p* < 0.001; Intermediate vs. High, *p* = 0.047
Lymphocyte (×10^9^/L)	1.9 (1.5–2.3)	1.6 (1.2–2.0)	1.3 (1.0–1.7)	<0.001	Low vs. Intermediate, *p* = 0.041; Low vs. High, *p* < 0.001; Intermediate vs. High, *p* = 0.044
SII	520 (420–650)	720 (580–890)	1020 (880–1280)	<0.001	Low vs. Intermediate, *p* < 0.001; Low vs. High, *p* < 0.001; Intermediate vs. High, *p* = 0.002
PLR	130 (105–165)	155 (120–190)	180 (140–220)	0.032	Low vs. High, *p* = 0.034
Ejection fraction (%)	55.8 ± 7.6	52.1 ± 8.4	48.3 ± 9.2	0.004	Low vs. High, *p* = 0.003
Cr/Cl (mL/min)	84.2 ± 20.5	77.6 ± 21.8	69.1 ± 23.2	0.018	Low vs. High, *p* = 0.016

Data are presented as mean ± standard deviation, median (interquartile range), or *n* (%), as appropriate. Overall *p*-values were calculated using one-way analysis of variance for normally distributed continuous variables, the Kruskal–Wallis test for non-normally distributed continuous variables, and the chi-square test or Fisher’s exact test for categorical variables, as appropriate. Significant overall comparisons were followed by Tukey’s post hoc test after analysis of variance, Dunn’s test with Holm adjustment after the Kruskal–Wallis test, or Holm-adjusted pairwise categorical comparisons, as appropriate. Only statistically significant adjusted pairwise comparisons are shown; “—” indicates that the overall comparison was not significant and pairwise testing was therefore not performed. SII, systemic immune-inflammation index; PLR, platelet-to-lymphocyte ratio; Cr/Cl, creatinine clearance.

**Table 3:** Reduced multivariable linear regression models for CCTA-derived SYNTAX I score.

Model/Variable	B	SE	95% CI for B	Standardized β	*p*-Value	VIF
**Model 1: Age, EF, Cr/Cl, and SII**						
Age, per year	0.14	0.058	0.03 to 0.25	0.16	0.019	1.14
Ejection fraction, per 1%	−0.24	0.096	−0.43 to −0.05	−0.22	0.014	1.20
Creatinine clearance, per 1 mL/min	−0.073	0.033	−0.139 to −0.007	−0.17	0.029	1.22
SII, per doubling	6.10	1.30	3.52 to 8.68	0.46	<0.001	1.12
**Model 2: Age, EF, Cr/Cl, and PLR**						
Age, per year	0.18	0.064	0.05 to 0.31	0.20	0.006	1.16
Ejection fraction, per 1%	−0.29	0.105	−0.50 to −0.08	−0.27	0.007	1.21
Creatinine clearance, per 1 mL/min	−0.082	0.036	−0.154 to −0.010	−0.19	0.025	1.24
PLR, per doubling	2.35	1.43	−0.49 to 5.19	0.15	0.104	1.08
**Model performance**						
**Model**	**R^2^**	**Adjusted R^2^**		**RMSE**	**Overall model test**	
Model 1	0.48	0.46		7.08	F(4,88) = 20.31; *p* < 0.001	
Model 2	0.35	0.32		7.91	F(4,88) = 11.85; *p* < 0.001	

B, unstandardized regression coefficient; SE, standard error; CI, confidence interval; β, standardized regression coefficient; VIF, variance inflation factor; RMSE, root mean squared error; EF, ejection fraction; Cr/Cl, creatinine clearance; SII, systemic immune-inflammation index; PLR, platelet-to-lymphocyte ratio.

**Table 4:** Reduced Firth penalized logistic regression models for high SYNTAX score (≥33).

Model/Variable	Penalized OR	Profile-Likelihood 95% CI	*p*-Value
**Model 1: Age and SII**			
Age, per year	1.04	1.00–1.09	0.048
SII, per 100-point increase	1.15	1.07–1.25	<0.001
**Model 2: Age and PLR**			
Age, per year	1.05	1.01–1.10	0.021
PLR, per 10-point increase	1.06	1.01–1.12	0.026

Firth penalized logistic regression was used because only 18 patients had a high SYNTAX score. Each model contained two prespecified parameters, corresponding to nine events per parameter. SII and PLR were evaluated in separate models because of their mathematical overlap. OR, odds ratio; CI, confidence interval; SII, systemic immune-inflammation index; PLR, platelet-to-lymphocyte ratio.

## Data Availability

The original contributions presented in this study are included in the article/Supplementary Material. Further inquiries can be directed to the corresponding author.

## Supplementary Materials

The following supporting information can be downloaded at: https://www.mdpi.com/article/10.3390/diagnostics16152417/s1, Table S1: Comparison of patients included in the analysis and patients excluded because of <50% coronary stenosis; Table S2: Distribution of cardiovascular medication use across CCTA-derived SYNTAX I score categories; Table S3: Sensitivity analysis according to the interval between blood sampling and CCTA; Table S4: Bootstrap internal validation of the reduced Firth logistic regression models; Table S5: Two-by-two classification tables for SII and PLR at the reported cut-offs.

## Abbreviations

The following abbreviations are used in this manuscript:

CAD	Coronary Artery Disease
CCTA	Coronary Computed Tomography Angiography
SYNTAX	Synergy Between PCI with Taxus and Cardiac Surgery
SII	Systemic Immune-Inflammation Index
PLR	Platelet-to-Lymphocyte Ratio
PCI	Percutaneous Coronary Intervention
CABG	Coronary Artery Bypass Grafting
ROC	Receiver Operating Characteristic
AUC	Area Under the Curve
OR	Odds Ratio
CI	Confidence Interval
SD	Standard Deviation
IQR	Interquartile Range
VIF	Variance Inflation Factor
CT	Computed Tomography
PACS	Picture Archiving and Communication System
