# Supplementary material for: Association of the Systemic Immune-Inflammation Index with CCTA-Derived SYNTAX Scores in Patients with Obstructive Coronary Artery Disease
Source: Diagnostics (Basel). 2026 Jul 31;16(15):2417. doi: 10.3390/diagnostics16152417 (PMC13465944; doi:10.3390/diagnostics16152417)
Supplement: Supplementary file 1 [file diagnostics-16-02417-s001.zip › diagnostics-4397797-supplementary.pdf]

Supplementary Table S1. Comparison of patients included in the analysis and patients excluded because of <50% coronary stenosis.

Variable	Obstructive CAD (≥50% stenosis), n = 93	Non-obstructive CAD (<50% stenosis), n = 68	p-value
Age (years)	62.4 ± 10.8	57.8 ± 11.4	0.011
Male sex, n (%)	64 (68.8)	39 (57.4)	0.135
Hypertension, n (%)	58 (62.4)	34 (50.0)	0.117
Diabetes mellitus, n (%)	36 (38.7)	17 (25.0)	0.068
Current smoking, n (%)	41 (44.1)	24 (35.3)	0.261
Dyslipidemia, n (%)	49 (52.7)	29 (42.6)	0.208
COPD, n (%)	14 (15.1)	7 (10.3)	0.376
Peripheral vascular disease, n (%)	11 (11.8)	4 (5.9)	0.200
Hemoglobin (g/dL)	13.6 ± 1.5	13.8 ± 1.4	0.387
Hematocrit (%)	40.8 ± 4.3	41.5 ± 4.1	0.296
Platelet count ($\times10^9$/L)	256 (210–305)	238 (200–285)	0.099
Neutrophil count ($\times10^9$/L)	4.9 (3.8–6.2)	4.1 (3.2–5.1)	0.003
Lymphocyte count ($\times10^9$/L)	1.7 (1.3–2.2)	1.9 (1.5–2.4)	0.069
SII	650 (480–890)	520 (390–700)	0.003
PLR	150 (115–195)	126 (100–164)	0.006
Ejection fraction (%)	52.6 ± 8.9	56.4 ± 7.5	0.004
Creatinine clearance (mL/min)	78.4 ± 22.1	86.7 ± 21.5	0.018

Data are presented as mean ± standard deviation, median (interquartile range), or number (percentage), as appropriate. Continuous variables were compared using the independent-samples t-test or Mann–Whitney U test according to their distributions. Categorical variables were compared using the chi-square test or Fisher's exact test, as appropriate. CAD, coronary artery disease; COPD, chronic obstructive pulmonary disease; SII, systemic immune-inflammation index; PLR, platelet-to-lymphocyte ratio.

Supplementary Table S2. Distribution of cardiovascular medication use across CCTA-derived SYNTAX I score categories.

Medication	Overall (n = 93)	Low (n = 48)	Intermediate (n = 27)	High (n = 18)	p-value
Statin, n (%)	63 (67.7)	29 (60.4)	20 (74.1)	14 (77.8)	0.286
Antiplatelet agent, n (%)	59 (63.4)	26 (54.2)	19 (70.4)	14 (77.8)	0.140
ACE inhibitor, n (%)	43 (46.2)	19 (39.6)	14 (51.9)	10 (55.6)	0.401
Beta-blocker, n (%)	49 (52.7)	22 (45.8)	16 (59.3)	11 (61.1)	0.390

Values are presented as n (%). Comparisons were performed using the chi-square test. CCTA, coronary computed tomography angiography; SYNTAX, Synergy Between PCI With Taxus and Cardiac Surgery; ACE, angiotensin-converting enzyme.

Supplementary Table S3. Sensitivity analysis according to the interval between blood sampling and CCTA.

Analysis population	n	High-SYNTAX cases, n	Inflam. index	SYNTAX I, r (p-value)	SYNTAX II PCI, r (p-value)	SYNTAX II CABG, r (p-value)
Full cohort	93	18	SII	0.59 (<0.001)	0.58 (<0.001)	0.55 (<0.001)
(±15 days)			PLR	0.34 (0.002)	0.29 (0.008)	0.27 (0.014)
Same-day	41	8	SII	0.62 (<0.001)	0.59 (<0.001)	0.56 (<0.001)
measurements			PLR	0.31 (0.048)	0.28 (0.076)	0.25 (0.115)
Measurements	72	14	SII	0.60 (<0.001)	0.58 (<0.001)	0.55 (<0.001)
within ≤5 days			PLR	0.33 (0.005)	0.30 (0.010)	0.28 (0.017)

The absolute interval between blood sampling and CCTA was calculated for each patient. When multiple eligible complete blood count measurements were available, the measurement closest to CCTA was selected. The same-day subgroup was included within the ≤5-day subgroup. Correlations were evaluated using the same correlation method applied in the primary analysis. CCTA, coronary computed tomography angiography; SII, systemic immune-inflammation index; PLR, platelet-to-lymphocyte ratio; PCI, percutaneous coronary intervention; CABG, coronary artery bypass grafting.

Supplementary Table S4. Bootstrap internal validation of the reduced Firth logistic regression models.

Model/performance measure	Apparent estimate	Estimated optimism	Optimism-corrected estimate	Bootstrap 95% CI
**Model 1: Age and SII**				
AUC	0.86	0.04	0.82	0.72–0.90
Calibration slope	1.00	0.17	0.83	0.55–1.15
Brier score	0.105	−0.011	0.116	0.087–0.151
**Model 2: Age and PLR**				
AUC	0.75	0.05	0.70	0.57–0.82
Calibration slope	1.00	0.25	0.75	0.43–1.11
Brier score	0.131	−0.013	0.144	0.109–0.179

Internal validation was performed using 1000 bootstrap resamples. In each bootstrap sample, the complete modeling procedure was repeated, and model performance was evaluated in both the bootstrap sample and the original dataset. Estimated optimism was calculated as the mean difference between bootstrap-sample and test-sample performance. Optimism-corrected estimates were calculated by subtracting estimated optimism from the apparent estimates. For the Brier score, negative optimism indicates that prediction error was greater during bootstrap testing than in the apparent analysis; therefore, correction resulted in a higher Brier score. Higher AUC values and calibration slopes closer to 1 indicate better performance, whereas lower Brier scores indicate lower overall prediction error. AUC, area under the receiver operating characteristic curve; CI, confidence interval; SII, systemic immune-inflammation index; PLR, platelet-to-lymphocyte ratio.

Supplementary Table S5. Two-by-two classification tables for SII and PLR at the reported cut-offs.

A. SII cut-off ≥850

SII classification	High SYNTAX (≥33)	Low/intermediate SYNTAX (<33)	Total
SII ≥850	15 (TP)	18 (FP)	33
SII <850	3 (FN)	57 (TN)	60
Total	18	75	93

B. PLR cut-off ≥165

PLR classification	High SYNTAX (≥33)	Low/intermediate SYNTAX (<33)	Total
PLR ≥165	12 (TP)	28 (FP)	40
PLR <165	6 (FN)	47 (TN)	53
Total	18	75	93

TP, true positive; FP, false positive; FN, false negative; TN, true negative; SII, systemic immune-inflammation index; PLR, platelet-to-lymphocyte ratio.
